# Zaira Cintra Vidal: a trajectory of innovation in Brazilian nursing education

**DOI:** 10.1590/0034-7167-2024-0328

**Published:** 2026-02-23

**Authors:** Mayki Bruno dos Santos Gonçalves, Thiago Augusto Soares Monteiro da Silva, Margareth Teixeira de Souza de Almeida, Camila Pureza Guimarães da Silva, Antonio José de Almeida, Tânia Cristina Franco Santos

**Affiliations:** 1 Universidade Federal do Rio de Janeiro. Rio de Janeiro, Rio de Janeiro, Brazil.

**Keywords:** Nursing, History of Nursing, Education, Nursing, Biography, Diffusion of Innovation

## Abstract

**Objectives:**

to analyze the contributions of Zaira Cintra Vidal’s academic-professional trajectory to innovation in Brazilian nursing education.

**Methods:**

a historical, qualitative study. Direct historical sources, such as written and iconographic documents, were used.

**Results:**

Zaira Contra Vidal led innovative achievements through scientific production published in the journal and authorship of textbooks, holding conferences and reorganizing healthcare services.

**Final Considerations:**

Zaira Cintra Vidal innovated the profession. The contributions of this innovation mark many gains in the present time, which can be exemplified by the uninterrupted conferences, the solidity of Revista Brasileira de Enfermagem and the Associação Brasileira de Enfermagem sections in the different states of Brazil.

## INTRODUCTION

Zaira Cintra Vidal’s professional life trajectory highlights her contribution to nursing education in Brazil, evidenced by her work in training nurses, as well as in the recognition of the importance of research for the development of the profession, through her publications in the form of books and articles in the journal *Annaes de Enfermagem* (current *Revista Brasileira de Enfermagem* (REBEn)). Zaira also records her honorable participation in *Associação Brasileira de Enfermeiras Diplomadas* (ABED), today called *Associação Brasileira de Enfermagem* (ABEn), as she was president of the association for two consecutive terms, namely: 1943-1945 and 1945-1947^(1,2)^.

Regarding nursing education in Brazil, in the first half of the 20th century, more precisely since 1923, with the inauguration of *Escola de Enfermeiras do Departamento Nacional de Saúde Pública*, currently *Escola de Enfermagem Anna Nery* (EEAN), the Anglo-American nursing model was implemented, which corresponded to the transposition of the Nightingale model to Brazil, adapted to the United States, almost half a century ago. On June 15, 1931, Decree 20,109, in its Article 2, determined EEAN as the official standard school in the country. This decree was in force until 1949^(3)^. Zaira Cintra Vidal is a graduate of this school, having been awarded a scholarship for a graduate course abroad. In addition to her student life, at the aforementioned school, she carried out important activities in favor of training nurses and preparing future nursing professors^(1,2)^.

Thus, the research question is: how did Zaira Cintra Vidal’s academic-professional trajectory impact nursing education in Brazil?

This study is justified by the fact that it deals with the successful academic and professional trajectory of a non-white nurse in the Brazilian context in the first half of the 20th century. Certainly, less than 50 years after the abolition of slavery, the marks left on Brazilian society were still indelible. Furthermore, it was a period in which the differences between males and females were legitimized, even by law. For instance, married women would only cease to be considered relatively incapable in 1962 (Law 4,121)^(4)^. Before the law, under the protection of her husband, she could not sign documents, receive inheritance, travel or even work. Therefore, such asymmetries between males and females were reproduced in different social spaces. Thus, the contribution of this study lies in bringing to light the legacy of a nurse^(5)^ who occupied positions of power and prestige both in training and care spaces, in addition to occupying the presidency of an organizational entity of the profession.

The time frame covers from 1926 to 1956. The initial corresponds to the year of Zaira Cintra Vidal’s graduation as a nurse at EEAN, and the end marks the year of graduation of students of the first graduate course at *Escola de Enfermeiras Rachel Haddock Lobo* (EERHL), entitled “nursing professor course”, which was inaugurated during Zaira Cintra Vidal’s term as director of EERHL (1944-1954).

## OBJECTIVES

To analyze the contributions of Zaira Cintra Vidal’s academic-professional trajectory to innovation in Brazilian nursing education.

## METHODS

### Ethical aspects

The research was approved by the EEAN/*Instituto de Atenção à Saúde São Francisco de Assis/Universidade Federal do Rio de Janeiro* (UFRJ) Research Ethics Committee. The historical sources are in the institutional custody of the EEAN Documentation Center (CDOC/EEAN/UFRJ) and the *Centro de Memória Nalva Pereira Caldas* (CMNPC) of the *Universidade do Estado do Rio de Janeiro* School of Nursing (UERJ/NUR). With this, the consent of both institutions was requested, which agreed to conduct the research. The Informed Consent Form does not apply to this research, since it deals with documentary and iconographic sources. The risks associated with the study are linked to the virtual environment. For this reason, data was stored in physical sources instead of being stored in online platforms or in the “cloud”.

### Study design

As this is a qualitative study, COnsolidated criteria for REporting Qualitative research (COREQ)^(6)^ were used to guide the methodological steps.

### Data source

The documentary universe was supported by written and iconographic/photographic sources, under the custody of CDOC/EEAN/UFRJ, CMNPC of UERJ/NUR and the Brazilian National Digital Library Digital Newspaper Library, which is part of *Fundação Biblioteca Nacional*, the authors’ own collection and in online databases.

### Data collection and organization

Data collection took place from May 15 to May 30, 2024. Textual sources were selected, digitized, and classified. The documentary *corpus* was established based on “criticism of adequacy”, which includes the following criteria: relevance; sufficiency; exhaustiveness; representativeness; homogeneity; and *corpus* organization by sector^(7)^. Based on the aforementioned criticism, the documentary *corpus* was constituted as follows: 18 written sources, 13 written documents, four journalistic articles and one photograph.

### Data analysis

The analysis of written documents and photographs was linked to the historical context of society at the time. With regard to the photographic text, in order to see beyond the fixed visibility, iconographic analysis was used, which consists of decoding the photographic record’s external reality, and iconological interpretation, which consists of understanding the record’s internal reality^(8)^. After this triangulation, the following categories of analysis emerged: Zaira Cintra Vidal’s academic-professional career; and Innovations for training nurses.

## RESULTS

Zaira Cintra Vidal, daughter of Eugenia da Silva Cintra Vidal and Amando de Araujo Cintra Vidal Junior, was born in Rio de Janeiro, then the capital of the country, on May 5, 1903^(9)^. The space designated for information about her race on the birth certificate is dashed. At the time of her birth, it was a form used to designate non-white people.

On March 19, 1924, at the age of 20, Zaira enrolled in the nursing course at the Brazilian National Department of Public Health school of nursing. On her application form, she declared that she was single and Catholic^(10)^. She also stated that she had ten years of schooling and that she had never worked in the nursing field^(10)^. Classes began on March 31, 1924. She graduated on August 6, 1926^(11)^. Soon after, still in August, she was appointed to the position of hygiene visitor, and on January 7, 1927, she was appointed public health nurse.

Given her performance, she was invited by the Rockefeller Foundation to continue her studies abroad. Thus, she went to the United States to complete a two-year refresher course (1927 to 1929). This course, taught at the Philadelphia General Hospital, Philadelphia Contagious Disease Hospital and Teachers College of Columbia University in the City of New York, aimed to prepare nurses in instruction and administration of nursing schools^(1)^.

Upon returning to Brazil in 1929, she was appointed head nurse at EEAN (currently, this position corresponds to that of undergraduate course coordinator), where she taught the following subjects: Ethics, History of Nursing, Nursing Techniques, Bandage Techniques, Personal Hygiene, Drugs and Solutions, and Techniques. Concerning the first nursing journal in Brazil, i.e., *Annaes de Enfermagem*, created in 1932, Zaira held the position of editor-proofreader and was responsible for the section called “Student Pages”. The following year, due to the death of Rachel Haddock Lobo, she became editor-in-chief, remaining there until 1938. In this journal, Zaira published several texts relevant to the profession, which encompassed assistance, teaching and management. Chart 1 presents the aforementioned publications:

**Chart 1:** Zaira Cintra Vidal’s publications on Annaes de Enfermagem from 1932 to 1941

Year	Nº	Title
1932	1	*Como pode as chefes de enfermarias cooperar para auxiliar a instrutora e como utilizar os casos e a prática para facilitar o ensino*
1934	3	*O triangulo da enfermeira*
1934	3	*A margem dos testes - análise dos vários tipos de testes*
1934	4	*O ensino e o uso do lesson plan*
1934	5	*O caso de estudo*
1935	6	*Enfermagem em caso de artrite*
1935	6	*Tradução do American Journal Nursing: como devemos avaliar a qualidade do serviço de enfermagem?*
1935	7	*Enfermagem em febre tifóide*
1937	9	*O trabalho pratico nas enfermarias*
1937	10	*Apanhados de técnica [1ª parte]*
1937	11	*Apanhados de técnica – Grupo dos Rubefacientes [2ª parte]*
1938	12	*Apanhados de técnica - Grupo dos Rubefacientes (uso das ventosas secas) [3ª parte]*
1941	17	*Apanhados de técnica – Aplicações locais frias*

*Source: EEAN Documentation Center*.

Zaira was very loved and recognized by students of EEAN, and was even honored by them in prose^(12)^:

I am sure that

If she did not exist

Escola Anna Nery would not it be the one,

The Standard School^(12)^

During her teaching career, Zaira observed the difficulties students had in developing nursing techniques and decided to write a book inspired by the Philadelphia General Hospital’s “Nursing Procedures” with adaptations for Brazilian nursing. Zaira made efforts to describe all the techniques of modern nursing, observing their objectives and scientific principles. The book was written based on the “Curriculum of School of Nursing”. It is worth noting that the work represents the first work on nursing techniques in Portuguese to be used by all nursing professionals in the country^(13)^.

**Figure 1:**
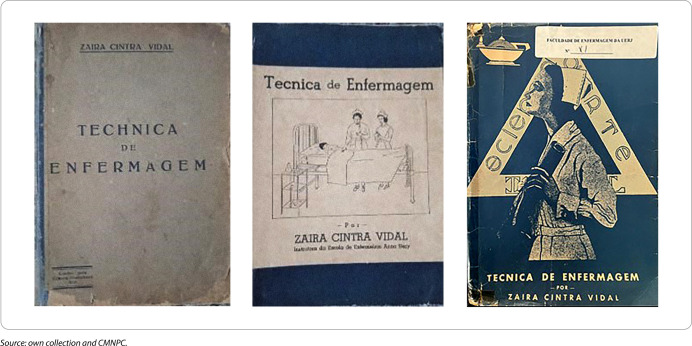
Nursing techniques book covers in ascending order of their editions

Zaira published three books, such as *“Technica de Enfermagem”, “Drogas e Soluções em dez aulas”* and *“Técnicas de Ataduras”*, respectively, in 1933, 1934 and 1938. These works corresponded to some subjects she taught at EEAN. Figure 1 shows the books *“Técnica de Enfermagem”* with their different covers and editions.

The second cover, corresponding to the 3rd edition (1942), features a drawing of two nurses in uniform and wearing caps caring for a patient in bed^(13)^. On the third cover, corresponding to the 5th, 6th and 7th editions^(14)^, the book has a navy blue cover, with the nurse’s lamp in the upper left corner. The nurse, in uniform and wearing a cap, is in the center of the composition, practically dividing the cover of the book. Her gaze is directed towards the lamp, while she holds a diploma holder. One can also see a triangle with the following inscription: “science, art and ideal”.

The meaning of the triangle is explained by Zaira in her publication entitled *“O Triângulo da Enfermeira”*. In this publication, she highlights the modern nurse’s attributes, highlighting discussions on the concepts as follows: the “ideal”, the basis for any student to choose the profession and the strength to overcome, highlighting Anna Nery’s contribution; the “science”, emphasizing that it was provided through training based on the Nightingale model and that it must be at the same intellectual level as the doctor; and the “art” of subjective practical application of scientific knowledge for each patient^(15)^.

The book is dedicated to Rachel Haddock Lobo and has a preface written by her. In the preface, Rachel mentions Professor Zaira’s intelligence and her importance for training nurses. The last paragraph summarizes Zaira’s transcultural perspective, ahead of her time in the production of Brazilian nursing science by comparing the Philadelphia General Hospital technique with the French techniques, adapting them to the Brazilian reality^(13)^.

When teaching students, Zaira was concerned with preparing nurses to work as professors and, in this regard, she used the following publications from the journal: *“A margem dos testes - análise dos vários tipos de testes”*, presenting the importance of carrying out a good test to be given to students^(16)^; *“O ensino e o uso do lesson plan”*, in which Zaira explained how to prepare a lesson plan^(17)^; and “O caso de estudo”, teaching how to work with clinical cases as a teaching-learning strategy^(18)^.

From 1929 to 1938, Zaira participated in examination boards for candidates, with a view to entering EEAN as a student. She also participated in the Inter-American Conference on Mental Hygiene, which took place in 1935. At this event, Zaira presented the “Nursing Aptitude Test” from Vanderbilt University, United States, which she adapted to Brazil^(19)^. Furthermore, she also participated in examination boards for practical nurses^(11)^.

**Figure 2:**
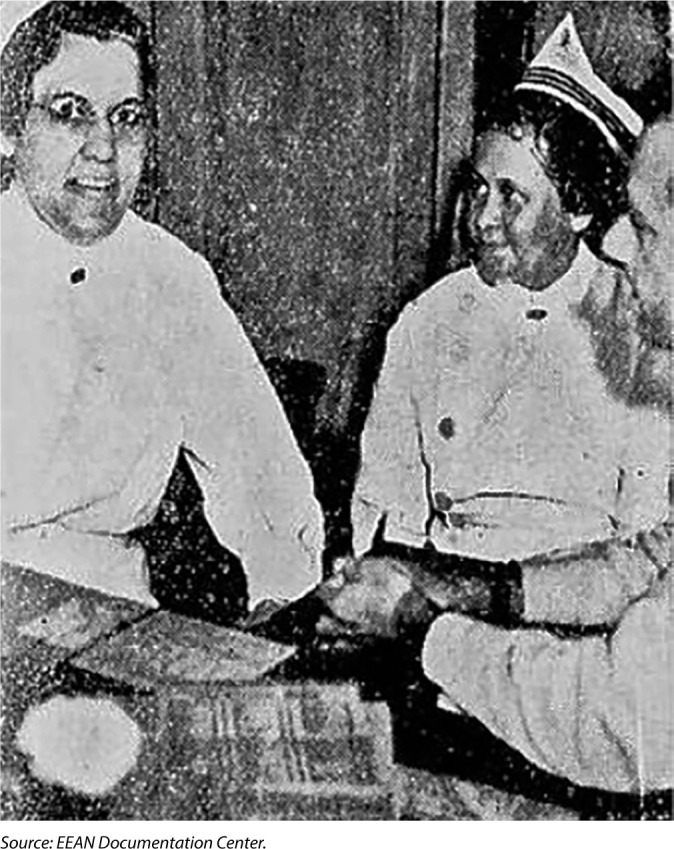
Bertha Pullen and Zaira Cintra Vidal on the cover of the newspaper “A Noite”

In 1938, the newspaper “A Noite” published a news article entitled *“Na Officina da Bondade”*. On the cover of the newspaper, Zaira Cintra Vidal is next to Bertha Pullen, an American nurse, then director of EEAN, in her second term (1934-1938). In the news article, Zaira is presented as a technical instructor at EEAN, and the content refers to the achievements of EEAN students and professors in assisting patients at *Hospital São Francisco de Assis*, highlighting that the education offered at the school is of quality, with emphasis on qualified teaching staff, which even included a professor with a graduate degree from the United States, such as Zaira Cintra Vidal. The school’s adequate facilities also stood out^(20)^. Figure 2 illustrates the material.

That same year, in May, Bertha Pullen appointed Zaira to be assistant director. In addition to this, from October to December, Zaira as school acting director. In this capacity, Zaira received thanks from the dean, Professor Raul Leitão da Cunha, for her services to the university in Brazil^(11)^.

In assistance, on June 15, 1941, Zaira began the organization of the hospitals of the General Department of Health and Assistance by agreement between the General Secretary of Health and Assistance, Jesuíno Carlos de Alburquerque, and the director of EEAN. Moreover, she directed the nursing services at the Emergency Room, Carlos Chagas, Getúlio Vargas and Jesus hospitals, which were institutions belonging to the Department of Hospital Assistance of the Federal District City Hall^(11)^.

On September 15, 1941, Zaira became a member of a committee of nurses, whose purpose was to study the minimum program for nursing schools. This committee was also tasked with studying the request for equalization of three schools: *Escola de Enfermeiras Luíza de Marillac* (created in 1939); *Escola de Enfermagem Carlos Chagas* (created in 1933); and *Escola Paulista de Enfermagem* (created in 1939). The following year, on May 25, precisely, Zaira ceased to be a member of the aforementioned committee, as she was placed at the disposal of the Government of the State of São Paulo (Process 6,177/42) of the Budget Division of the Ministry of Education and Health Department of Health^(11)^.

During World War II, Zaira Cintra Vidal promoted several war relief courses. These courses were reported in major newspapers, with notable participation of Zaira Cintra Vidal, a professor at EEAN (the official standard school at the time). Therefore, Zaira was a reference in nursing care in times of crisis^(11,21)^.

In May 1943, Zaira Cintra Vidal was invited by Claire Louise Kienniger, the first former director of EEAN and then director of nursing for the special public health service, to teach a course on emergency nursing, as well as assist in the sanitation of Vale do Rio Doce, organized by Kienniger. This work resulted in the organization of a school that was named *Curso de Emergência de Guerra Alda dos Santos Neves* ^(11)^.

Zaira’s work on different fronts allowed her to gain recognition for her competency and leadership, as she as president of ABED, now ABEn, for two consecutive terms (1943 to 1945 and 1945 to 1947)^(22,23)^.

In 1943, Zaira traveled abroad to visit hospitals, schools and universities. She visited New York Hospital, including: National Nursing Headquarters; Skidmore College e New York Post-Graduate and Medical School Hospital; Teachers College, Columbia University; Division of Nursing, Department of Hospitals; Yale University School of Nursing; State Board of Nurses Examiners Albany; Toronto University School of Nursing; Western Reserve University School of Nursing. However, she stopped visiting Philadelphia General Hospital due to illness^(24)^.

In 1943, the front page of the newspaper *“A Noite”*, from Rio de Janeiro, published the following news: *“Uma Escola de Enfermeiras em cada Estado”* (A School of Nursing in Every State). This publication was obtained through an interview given by Zaira Cintra Vidal, in her capacity as president of ABED. The text highlights Zaira’s speech regarding the fact that Brazil only has five nursing schools, which graduate an average of 35 nurses annually, and Zaira’s visits to hospitals and schools, with the aim of studying new methods of nursing education^(25)^.

It is worth noting that Zaira Cintra Vidal, when highlighting the existence of only five schools, considered only those that were equivalent to the standard teaching model in the country. Nevertheless, Zaira was present in other nursing education institutions as well as participating in assessment panels for practical nurses and validation of diplomas^(21,24)^.

The details of Zaira’s visits to hospitals and nursing schools abroad are included in the “Report of Studies Made in North America”, completed in 1944. This includes all the relevant points of each institution visited. Certainly, the knowledge acquired by Zaira constituted important content for the formulation of Law 775 of August 6, 1949, which dealt with nursing education in the country. This subject was the subject of discussion at meetings of directors of nursing schools, where Zaira participated as president of ABED, also being a school director^(24)^.

During Zaira’s term as president of ABED, the first Brazilian National Nursing Congress (today called the Brazilian Nursing Congress) was held in São Paulo from March 17 to 22, 1947. The organization of the event’s schedule was the responsibility of Zaira Cintra Vidal and nurse Ella Hasenjaegem, as president of the organizing committee, in addition to Edith de Magalhães Fraenkel and Sister of Charity Marie Domineuc^(26)^.

Zaira Cintra Vidal, from 1943 to 1947, was also responsible for creating and implementing the project to create EERHL, the current UERJ School of Nursing. It is worth mentioning that the school was created in 1944, but the inauguration took place in 1948. The equivalence took place in 1949. Zaira Cintra Vidal held the position of director from 1944 to 1954^(22)^.

**Figure 3:**
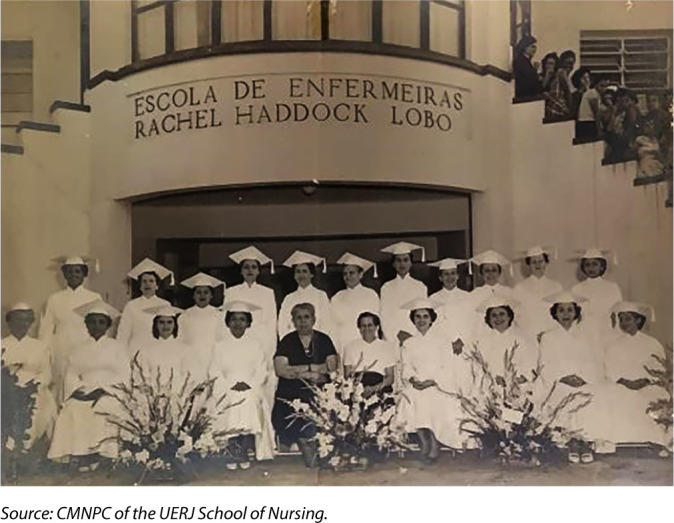
Graduation of the graduate course in nursing professor at Escola de Enfermeiras Rachel Haddock Lobo

In addition to the undergraduate nursing course, Zaira was a pioneer in coordinating the first two graduate courses for nursing professors, lasting two years (1955 to 1956), and for hospital management, lasting one year (1956)^(22)^. Figure 3 shows the graduation photograph of the first class of the EERHL nursing professor training course in 1956.

The 19 graduates are in uniform and wear caps. The photographic composition includes 21 women, all nurses. In the foreground of the composition, ten nurses are seated, with the fifth person from left to right being Sylvia Arcoverde Albuquerque Maranhã, in civilian attire, then director of EERHL, who is on her left with Zaira Cintra Vidal, also in civilian attire^(27)^.

Zaira Cintra Vidal also worked with inmates at *Presidio Frei Caneca* to promote education, as she shared ideas against illiteracy, adopting the Enlightenment maxim that emerged in the 19th century, which was “to open schools is to close prisons”. Therefore, Zaira was a nurse present in different scenarios of Brazilian society.

## DISCUSSION

Zaira Cintra Vidal, born at the beginning of the 20th century, was a non-white woman whose social class was considered middle class^(28)^. During her academic career, she was successful in entering a nursing school where she was successful, having done graduate studies abroad and becoming a professor at that same school, in prominent positions. It is worth reflecting that less than half a century had passed since the abolition of slavery and, certainly, the consequences greatly affected those who were enslaved and their descendants. Therefore, Zaira’s inclusion in school and her successful trajectory demonstrate her significant capacity for study and work, since, at the beginning of the century, the school modeled after Nightingale ensured a good selection, admitting “girls from good families”, i.e., those with white skin, to characterize the image of the new profession in Brazilian society^(28,29)^.

The fact is that social mobility for the black and mixed-race population in the 1920s was difficult, because, given the conditions of rising families, they had little access to education, especially black women^(28)^. This situation resonates in the present day and was certainly much worse back then. In this context, bringing it to the present day, Zaira would be socially seen as brown. Thus, her knowledge and competence allowed her to have social mobility^(29)^.

In addition to her work as a professor, Zaira played an important role in the dissemination of scientific knowledge, through her significant participation in the creation of the *Annaes de Enfermagem* journal, from 1930 to 1932. With the death of Rachel Haddock Lobo, on September 26, 1933, Zaira Cintra Vidal, then vice-president of ABED, took over as editor-in-chief on October 23, 1933, by unanimous vote, at a general meeting of the association. On that same day, November 25 was set as the date for the release of the second issue. The first pages were dedicated to the memory of Rachel Haddock Lobo. At the end of 1938, on the occasion of a new election for the association, Zaira declared that she could no longer continue due to her numerous activities.

Zaira, while she was editor-in-chief of the journal, began her journey as a writer of nursing books. The books were about nursing techniques of the time. They provided detailed information on nursing procedures, in accordance with technical and scientific language. The procedures were listed in order of complexity, starting from the simplest to the most complex. It was therefore an educational book, easy to read and assimilate. At the end of each technique, there was a space called “notes”. This showed that Zaira was a person open to receiving contributions, in order to advance her reflections. This demonstrated her understanding that knowledge is something dynamic and that it should be shared horizontally^(30)^.

Thus, Zaira democratized knowledge with teaching material in Portuguese, giving access to people who did not understand English. Furthermore, it was a work based on the thinking of modern nursing. Regarding the publication of books, before those of Zaira Cintra Vidal, there was a book written by the doctor and army officer Getúlio F. dos Santos called “O livro do enfermeiro e da enfermeira”, which was intended for those who dedicated themselves to caring for the sick, in the exercise of work activities, as a nurse. The book was published in 1916^(31)^. Therefore, Zaira was the first Brazilian nurse to systematize nursing techniques in a book. Moreover, she transformed, with her scientific knowledge, the techniques for the Brazilian reality^(13)^.

Zaira’s book was dedicated and prefaced by Rachel Haddock Lobo. This strategy certainly gave the writer and her work visibility and credibility, since Rachel Haddock Lobo was a prestigious figure in education and health in Brazil, with outstanding performance in EEAN and ABED. It is worth mentioning that, on the occasion of her death, scientist Carlos Chagas gave a prestigious speech at her graveside, urging nurses to follow her example^(8)^.

It is important to highlight that Zaira Cintra Vidal’s graduate studies gave her the skills to take on leadership roles in education and health. However, despite her successful career at EEAN, she did not reach the position of school director, despite acting as interim director and managing the school, currently in a position corresponding to that of vice-director. However, at ABED, she was elected by her peers to the position of president for two consecutive terms.

In addition to publishing books, during her time at ABED, she created association sessions in Brazilian states where there were nursing schools. This approach was innovative for the profession, as it strengthened the association while ensuring the formation of groups capable of leading the profession, in order to advance the dissemination of knowledge and achievements for Brazilian nursing. Furthermore, it was during his administration that the first Brazilian National Nursing Congress was held, known today as the Brazilian Nursing Congress. Holding a congress at a national level symbolizes the establishment of a first intellectual space for the profession, providing exchange, dissemination of knowledge and visibility. Thus, Zaira’s avant-garde vision, in favor of the development of the profession, especially in education, brings to light, in the 1940s and 1950s, innovation for future nurses, since learning ways of caring involves study and research, i.e., “innovations/changes to be instituted”^(32)^.

It is worth noting that the congress and the journal represented the first intellectual spaces for the profession. In addition to contributing to exchange and advancement of knowledge, they provided visibility for the profession in Brazilian society, especially with regard to its connection with production of scientific knowledge, which is so necessary for advancement of nursing and promotion of safe care. Thus, Zaira was a leading figure of an innovative vision, in the sense of recognizing the importance of the production and dissemination of scientific knowledge about nursing and, consequently, health^(33)^. These achievements reverberate in the present time, when we come across the congress, now in its 74th version, and like the journal, with an important impact factor in the scientific community.

The recognition of Zaira’s work in healthcare, especially in organizing hospital health services and training nursing staff at the Rio de Janeiro city government, led her to be chosen to plan EERHL, which led her to the position of director. Thanks to all of Zaira’s professional competence, the school was brought up to standard in less than a year of operation^(34)^. At the time, Zaira opened the school’s doors to black girls, allowing social mobility for girls who faced a barrier to entering nursing education^(29)^.

In addition to training staff at the Federal District City Hall and the EERHL nursing course, in 1955, EERHL graduate courses were started in the American style, at a time when there was still no regulation in the country, which would only happen in the 1960s^(35)^. Therefore, here is its vanguard and the ability to innovate nursing training through graduate studies.

Therefore, perceiving innovation in nursing as synonymous with change, it is pertinent to state that Zaira Cintra Vidal innovated the profession, as she thought and acted beyond the traditional, the common, what was determining, towards the new^(36)^. It can be said that, in the first half of the 20th century, Zaira dared to be a visionary and leading figure of changes, mainly in nursing education linked to the nascent scientific production.

### Study limitations

The researchers were unable to contact oral sources who had contact with Zaira as family members, friends or students, which is a weakness in the bibliographic construction, as it could enrich the study with subjective details of Zaira’s actions and thoughts, making the study less linear. To this end, sources from different means of production and nature were triangulated to generate the results to be analyzed and interpreted.

### Contributions to nursing, health or public policy

The study contributes to preserving the memory of Zaira Cintra Vidal’s life story, an icon for nursing education and health, filling gaps in knowledge about the history of nursing, especially with regard to ABEn, for the consolidation of REBEn and for the regulation of nursing education in the country.

## FINAL CONSIDERATIONS

Zaira Cintra Vidal had an innovative role in Brazilian nursing. The contributions of this innovation mark many gains in the present time, which can be exemplified by the uninterrupted congresses, the solidity of REBEn and the sections of ABEn in the different states of Brazil.

Therefore, Zaira was a pillar of the profession in teaching, research, care and associations. Still as reflections of her work, Zaira leaves an important legacy for the female and “non-white” population, as it was called at the time, through the consecration of a successful trajectory through the positions held both at ABEn and at renowned nursing schools in the then federal district. Such actions reflect her capacity for study, work and strength to fight for the participation of women in important public spaces.

## Data Availability

The research data are available only upon request.
