# Supplementary material for: Zaira Cintra Vidal: a trajectory of innovation in Brazilian nursing education
Source: Rev Bras Enferm. 2026 Feb 23;79:e20240328. doi: 10.1590/0034-7167-2024-0328 (PMC12933936; doi:10.1590/0034-7167-2024-0328)
Supplement: Translation

# Translation: Zaira Cintra Vidal: uma trajetória de inovação na educação da enfermagem brasileira

**DOI:** 10.1590/0034-7167-2024-0328pt

**Authors:** Mayki Bruno dos Santos Gonçalves, Thiago Augusto Soares Monteiro da Silva, Margareth Teixeira de Souza de Almeida, Camila Pureza Guimarães da Silva, Antonio José de Almeida, Tânia Cristina Franco Santos

**Affiliations:** I Universidade Federal do Rio de Janeiro. Rio de Janeiro, Rio de Janeiro, Brasil.

**Keywords:** Enfermagem, História da Enfermagem, Educação em Enfermagem, Biografia, Difusão de Inovações

## Abstract

**Objetivos:**

analisar as contribuições da trajetória acadêmico-profissional de Zaira Cintra Vidal à inovação na educação da enfermagem brasileira.

**Métodos:**

estudo histórico, qualitativo. Fontes históricas diretas, como documentos escritos e iconográficos, foram utilizadas.

**Resultados:**

Zaira Contra Vidal protagonizou realizações inovadoras por meio de produção científica publicada na revista e autoria de livros didáticos, realização de congressos e reorganização de serviços assistenciais.

**Considerações Finais:**

Zaira Cintra Vidal inovou a profissão. As contribuições dessa inovação demarcam muitos ganhos no tempo presente, os quais podem ser exemplificados pelos congressos ininterruptos, pela solidez da Revista Brasileira de Enfermagem e pelas seções da Associação Brasileira de Enfermagem nos diferentes estados do Brasil.

## INTRODUÇÃO

A trajetória de vida profissional de Zaira Cintra Vidal evidencia sua contribuição à educação em enfermagem no Brasil, evidenciada na sua atuação na formação de enfermeiras, bem como no reconhecimento da importância da pesquisa para o desenvolvimento da profissão, por meio de suas publicações sob a forma de livros e publicação de artigos na revista Annaes de Enfermagem (atual Revista Brasileira de Enfermagem (REBEn)). Zaira também registra a honrosa participação na Associação Brasileira de Enfermeiras Diplomadas (ABED), hoje chamada de Associação Brasileira de Enfermagem (ABEn), pois exerceu a presidência da associação por dois mandatos consecutivos, quais sejam: 1943 -1945 e 1945-1947^(1,2)^.

No que se refere à educação em enfermagem no Brasil, na primeira metade do século XX, mais precisamente desde o ano de 1923, com a inauguração da Escola de Enfermeiras do Departamento Nacional de Saúde Pública, atual Escola de Enfermagem Anna Nery (EEAN), foi implantado o modelo anglo-americano de enfermagem, que correspondeu à transposição do modelo nightingaleano para o Brasil, adaptado aos Estados Unidos, há quase meio século. Em 15 de junho de 1931, o Decreto nº 20.109, em seu Artigo 2º, determinou a EEAN como escola oficial padrão no país. Esse decreto vigorou até 1949^(3)^. Zaira Cintra Vidal é egressa dessa escola, tendo sido laureada com bolsa para curso de pós-graduação no exterior. Além da vida estudantil, na citada escola, exerceu atividades de monta em prol da formação de enfermeiras e no preparo de futuras docentes de enfermagem^(1,2)^.

Assim, a questão de pesquisa é: como a trajetória acadêmico-profissional de Zaira Cintra Vidal impactou a educação em enfermagem no Brasil?

O presente estudo se justifica por se tratar da trajetória acadêmico-profissional exitosa de uma enfermeira não branca, no contexto brasileiro, na primeira metade do século XX. Certamente, decorridos menos de 50 anos da abolição da escravidão, as marcas na sociedade brasileira ainda eram indeléveis. Ademais, era um período em que as diferenças entre o masculino e o feminino eram legitimadas inclusive por força de lei. A título de exemplificação, a mulher casada só viria a deixar de ser considerada relativamente incapaz em 1962 (Lei nº 4.121)^(4)^. Antes da lei, tutelada pelo marido, não podia assinar documentos, receber herança, viajar e até mesmo trabalhar. Portanto, tais assimetrias entre o masculino e o feminino eram reproduzidas nos diferentes espaços sociais. Assim, a contribuição do estudo reside em trazer à lume o legado de uma enfermeira^(5)^ que ocupou posições de poder e prestígio tanto nos espaços de formação quanto nos espaços de assistência, além da ocupação de presidência em entidade organizativa da profissão.

O recorte temporal abrange o período de 1926 a 1956. O inicial corresponde ao ano da formatura de Zaira Cintra Vidal como enfermeira na EEAN, e o final demarca o ano da formatura das estudantes do primeiro curso de pós-graduação na Escola de Enfermeiras Rachel Haddock Lobo (EERHL), intitulado “curso de professor em enfermagem”, cuja inauguração ocorreu na gestão de Zaira Cintra Vidal na direção da EERHL (1944-1954).

## OBJETIVOS

Analisar as contribuições da trajetória acadêmico-profissional de Zaira Cintra Vidal à inovação na educação da enfermagem brasileira.

## MÉTODOS

### Aspectos éticos

A pesquisa foi aprovada pelo Comitê de Ética em Pesquisa da EEAN/Instituto de Atenção à Saúde São Francisco de Assis/Universidade Federal do Rio de Janeiro (UFRJ). As fontes históricas estão na guarda institucional do Centro de Documentação da EEAN (CDOC/EEAN/UFRJ) e do Centro de Memória Nalva Pereira Caldas (CMNPC) da Faculdade de Enfermagem da Universidade do Estado do Rio de Janeiro (ENF/UERJ). Com isso, foi solicitada a anuência de ambas as instituições, no qual aceitaram a realização da pesquisa. O Termo de Consentimento Livre e Esclarecido não se aplica a esta pesquisa, por se tratar de fontes documentais e iconográficas. Os riscos associados ao estudo estão vinculados ao ambiente virtual. Por essa razão, os dados foram armazenados em fontes físicas ao invés de serem armazenados em plataformas *online* ou na “nuvem”.

### Tipo de estudo

Por se tratar de estudo qualitativo foi utilizado o instrumento *COnsolidated criteria for REporting Qualitative research* (COREQ)^(6)^ para nortear os passos metodológicos.

### Fonte de dados

O universo documental foi abalizado com fontes escritas e iconográficas/fotográficas, sob a guarda do CDOC/EEAN/UFRJ, do CMNPC da ENF/UERJ e da Hemeroteca Digital da Biblioteca Nacional Digital, que faz parte da Fundação Biblioteca Nacional, acervo dos próprios autores e nas bases de dados *online*.

### Coleta e organização dos dados

A coleta de dados ocorreu de 15 de maio a 30 de maio de 2024. As fontes textuais foram selecionadas, digitalizadas e classificadas. O *corpus* documental foi estabelecido com base na “crítica da adequação”, que comporta os seguintes critérios: pertinência; suficiência; exaustividade; representatividade; homogeneidade; e organização do *corpus* por setor^(7)^. Com base na referida crítica, o *corpus* documental ficou assim constituído: 18 fontes escritas, sendo 13 documentos escritos, quatro matérias jornalísticas e uma fotografia.

### Análise dos dados

A análise dos documentos escritos e do fotográfico foi vinculada ao contexto histórico da sociedade da época. No que se refere ao texto fotográfico, no intuito de enxergar além da visibilidade fixada, foram utilizadas a análise iconográfica, que consiste na decodificação da realidade exterior do registro fotográfico, e a interpretação iconológica, que consiste na compreensão da realidade interior do registro^(8)^. Após essa triangulação, emergiram as seguintes categorias de análise: Percurso acadêmico-profissional de Zaira Cintra Vidal; e Inovações para a formação de enfermeiras.

## RESULTADOS

Zaira Cintra Vidal, filha de Eugenia da Silva Cintra Vidal e de Amando de Araujo Cintra Vidal Junior, nasceu no Rio de Janeiro, então capital do país, em 05 de maio de 1903^(9)^. O espaço destinado à informação de sua cor na certidão de nascimento está tracejado. Na época de seu nascimento, era uma forma utilizada para a designação de pessoas não brancas.

No dia 19 de março de 1924, com 20 anos de idade, Zaira inscreveu-se no curso de enfermeiras da escola de enfermeiras do Departamento Nacional de Saúde Pública. Em sua ficha de inscrição, declarou ser solteira e católica^(10)^. Também consta a informação de dez anos de escolaridade e que nunca havia trabalhado na área da enfermagem^(10)^. As aulas tiveram início em 31 de março de 1924. Sua diplomação ocorreu em 6 de agosto de 1926^(11)^. Logo depois, ainda em agosto, foi designada para exercer o cargo de visitadora de higiene, e em 7 de janeiro de 1927, foi nomeada enfermeira de saúde pública.

Em face do seu desempenho, foi convidada pela Fundação Rockfeller para prosseguir estudos fora do país. Assim, foi para os Estados Unidos para realização de um curso de aperfeiçoamento durante dois anos (1927 a 1929). Tal curso, ministrado no *Philadelphia General Hospital, Philadelphia Contagious Disease Hospital* e no *Teachers College* da *Columbia University in the City of New York*, tinha como objetivo preparar enfermeiras em instrução e administração de escolas de enfermagem^(1)^.

Ao regressar ao Brasil, em 1929, foi nomeada enfermeira chefe da EEAN (na atualidade, esse cargo corresponde ao de coordenador de curso de graduação), onde lecionou as seguintes disciplinas: Ética, História da Enfermagem, Técnicas de Enfermagem, Técnicas de Ataduras, Higiene Individual, Drogas e Soluções, e Técnicas. No que concerne ao primeiro periódico de enfermagem, no Brasil, ou seja, Annaes de Enfermagem, criada em 1932, Zaira ocupou o cargo de redatora-revisora e era responsável pela seção denominada “Páginas das estudantes”. No ano seguinte, devido à morte de Rachel Haddock Lobo, passou a ocupar o cargo de redatora-chefe, permanecendo até o ano de 1938. Nessa revista, Zaira publicou diversos textos relevantes para profissão, os quais englobavam a assistência, docência e gerência. O Quadro 1 apresenta as referidas publicações:

**Quadro 1:** Publicações de Zaira Cintra Vidal no Annaes de Enfermagem no período de 1932 a 1941

Ano	Nº	Título
1932	1	Como pode as chefes de enfermarias cooperar para auxiliar a instrutora e como utilizar os casos e a prática para facilitar o ensino
1934	3	O triangulo da enfermeira
1934	3	A margem dos testes - análise dos vários tipos de testes
1934	4	O ensino e o uso do *lesson plan*
1934	5	O caso de estudo
1935	6	Enfermagem em caso de artrite
1935	6	Tradução do *American Journal Nursing*: como devemos avaliar a qualidade do serviço de enfermagem?
1935	7	Enfermagem em febre tifóide
1937	9	O trabalho pratico nas enfermarias
1937	10	Apanhados de técnica [1ª parte]
1937	11	Apanhados de técnica – Grupo dos Rubefacientes [2ª parte]
1938	12	Apanhados de técnica - Grupo dos Rubefacientes (uso das ventosas secas) [3ª parte]
1941	17	Apanhados de técnica – Aplicações locais frias

*Fonte: Centro de Documentação da EEAN*.

Zaira era muito querida e reconhecida pelas alunas da EEAN, e inclusive foi por elas homenageada em prosa^(12)^:

Tenho pela certeza de que

Se ela não existisse

A Escola Anna Nery não seria a tal,

A Escola Padrão^(12)^

Em sua atuação na docência, Zaira observou as dificuldades das alunas no desenvolvimento das técnicas de enfermagem e decidiu escrever um livro, inspirado no “*Nursing Procedures*”, do *Philadelphia General Hospital*, com adaptações à enfermagem brasileira. Zaira empreendeu esforços no sentido de descrever todas as técnicas da enfermagem moderna, observando os seus objetivos e princípios científicos. A escrita do livro teve como base o “*Curriculum of School of Nursing*”. Destaca-se que a obra representa um primeiro trabalho sobre técnicas de enfermagem, em português, para ser utilizada por toda a enfermagem do país^(13)^.

Zaira publicou um total de três livros, como “Technica de Enfermagem”, “Drogas e Soluções em dez aulas” e “Técnicas de Ataduras”, respectivamente, nos anos de 1933, 1934 e 1938. Tais obras correspondiam a algumas disciplinas por ela lecionadas na EEAN. A Figura 1 apresenta os livros “Técnica de Enfermagem” com suas diferentes capas e edições.

**Figura 1:** As capas do livro técnicas de enfermagem em ordem crescente de suas edições

A segunda capa, referente à 3ª edição (1942), figura o desenho de duas enfermeiras uniformizadas e com touca cuidando de um paciente no leito^(13)^. Na terceira capa, correspondente à 5ª, 6ª e 7ª edição^(14)^, o livro possui uma capa azul marinho, tendo no canto superior esquerdo a lâmpada da enfermagem. A enfermeira uniformizada e com touca está no centro da composição, praticamente cindindo a capa do livro. Seu olhar está direcionado para a lâmpada, ao tempo em que segura um porta-diploma. Vê-se também um triângulo com a seguinte escrita: “ciência, arte e ideal”.

O significado do triângulo é explicado por Zaira em sua publicação intitulada “O Triângulo da Enfermeira”. Nesta publicação, ela destaca os atributos da enfermeira moderna, destacando as discussões sobre os conceitos da seguinte forma: o “ideal”, a base para qualquer estudante escolher a profissão e a força para vencer, destacando a contribuição de Anna Nery; a “ciência”, enfatizando que era proporcionada por meio de uma formação baseada no modelo de Nightingale e que deve estar no mesmo nível intelectual do médico; e a “arte” da aplicação prática dos conhecimentos científicos de forma subjetiva para cada paciente^(15)^.

O livro é dedicado à Rachel Haddock Lobo e por ela prefaciado. No prefácio, Rachel faz menção à inteligência da professora Zaira e sua importância para a formação de enfermeiras. O último parágrafo resume bem todo o olhar transcultural de Zaira, a frente de seu tempo na produção de ciência da enfermagem brasileira ao confrontar a técnica do *Philadelphia Geral Hospital* e das técnicas a francesa, adaptando para a realidade brasileira^(13)^.

No ensino das estudantes, Zaira preocupava-se em preparar enfermeiras para atuarem como docentes e, nesse intento, utilizou as seguintes publicações da revista: “A margem dos testes - análise dos vários tipos de testes”, apresentando a importância da realização de um bom teste a ser feito às estudantes^(16)^; “O ensino e o uso do *lesson plan*”, em que Zaira explanava sobre a forma de elaborar um plano de aula^(17)^; e “O caso de estudo”, ensinando a trabalhar com casos clínicos como estratégia de ensino-aprendizagem^(18)^.

No período de 1929 a 1938, Zaira participou de bancas examinadoras de candidatas, com vistas ao ingresso na EEAN na qualidade de aluna. Também participou da Conferência Interamericana de Higiene Mental, que ocorreu em 1935. Neste evento, Zaira apresentou o “Teste de Aptidão em Enfermagem” da Universidade de Vanderbilt, Estados Unidos, por ela adaptado ao contexto brasileiro^(19)^. Ademais, participou ainda de bancas examinadoras de enfermeiros práticos^(11)^.

No ano de 1938, foi publicada no jornal a “A Noite”, uma matéria jornalística intitulada “Na Officina da Bondade”. Na capa do jornal, Zaira Cintra Vidal está ao lado de Bertha Pullen, enfermeira norte-americana, então diretora da EEAN, em sua segunda gestão (1934-1938). Na matéria jornalística Zaira, é apresentada como instrutora técnica da EEAN, e o conteúdo refere-se aos feitos das alunas e professoras da EEAN na assistência aos pacientes do Hospital São Francisco de Assis, destacando que o ensino oferecido na escola é de qualidade, com destaque para o corpo docente qualificado que contava, inclusive, com professora pós-graduada nos Estados Unidos, como era o caso de Zaira Cintra Vidal. As adequadas instalações da escola também são ressaltadas^(20)^. A Figura 2 ilustra a matéria.

**Figura 2:** Bertha Pullen e Zaira Cintra Vidal na capa do jornal “A Noite”

Nesse mesmo ano, precisamente em maio, Bertha Pullen designou Zaira para exercer o cargo de assistente de diretora. Além disso, no período de outubro a dezembro, Zaira ocupou interinamente o cargo de diretora da escola. Nesse mister, Zaira recebeu agradecimentos do reitor, Professor Raul Leitão da Cunha, pelos serviços prestados à universidade do Brasil^(11)^.

Na assistência, em 15 de junho de 1941, Zaira iniciou a organização dos hospitais da Secretaria Geral de Saúde e Assistência, por entendimento entre o Secretário Geral de Saúde e Assistência, Jesuíno Carlos de Alburquerque, e a diretora da EEAN. Além disso, dirigiu os serviços de enfermagem nos hospitais Pronto-Socorro, Carlos Chagas, Getúlio Vargas e Jesus, que eram instituições pertencentes ao Departamento de Assistência Hospitalar da Prefeitura do Distrito Federal^(11)^.

Em 15 de setembro de 1941, Zaira passou a fazer parte de uma comissão de enfermeiras, com a finalidade de estudar o programa mínimo para escolas de enfermagem. Essa comissão foi encarregada, também, de estudar o pedido de equiparação de três escolas: Escola de Enfermeiras Luíza de Marillac (criada em 1939); Escola de Enfermagem Carlos Chagas (criada em 1933); e Escola Paulista de Enfermagem (criada em 1939). No ano seguinte, precisamente em 25 de maio, Zaira deixou de fazer parte da comissão supramencionada, pois foi ficou à disposição do Governo do Estado de São Paulo (Processo nº 6.177/42) da Divisão do Orçamento do Departamento de Saúde do Ministério da Educação e Saúde^(11)^.

Durante a Segunda Guerra Mundial, Zaira Cintra Vidal promoveu diversos cursos de socorro de guerra. Esses cursos foram noticiados em jornais de grande circulação, com o destaque de que contavam com a notória participação de Zaira Cintra Vidal, professora da EEAN (escola oficial padrão à época). Portanto, Zaira era referência em assistência de enfermagem em tempos de crise^(11,21)^.

Em maio de 1943, Zaira Cintra Vidal foi convidada por Claire Louise Kienniger, primeira ex-diretora da EEAN e então diretora de enfermagem do serviço especial de saúde pública, para ministrar curso de emergência em enfermagem, além de auxiliar no saneamento do Vale do Rio Doce, organizado por Kienniger. Esse trabalho resultou na organização de uma escola que tomou o nome de Curso de Emergência de Guerra Alda dos Santos Neves^(11)^.

A atuação de Zaira em diferentes frentes de trabalhos lhe permitiu reconhecimento de sua competência e liderança, pois exerceu a presidência da ABED, atual ABEn, por dois mandatos consecutivos (1943 a 1945 e 1945 a 1947)^(22,23)^.

Em 1943, Zaira viajou para o exterior, com vistas a realizar visitas a hospitais, escolas e universidades. Assim, esteve no *New York Hospital*, incluindo: *National Nursing Headquarters*; *Skidmore College* e *New York Post-Graduate and Medical School Hospital*; *Teachers College*, *Columbia University*; *Division of Nursing, Department of Hospitals*; *Yale University School of Nursing*; *State Board of Nurses Examiners Albany*; *Toronto University School of Nursing*; *Western Reserve University School of Nursing*. Porém, deixou de visitar apenas o *Philadelphia General Hospital* em virtude de adoecimento^(24)^.

Em 1943, a primeira página do jornal “A Noite”, do Rio de Janeiro, publicava a seguinte notícia: “Uma Escola de Enfermeiras em cada Estado”. Essa publicação foi obtida por meio de entrevista concedida por Zaira Cintra Vidal, na qualidade de presidente da ABED. O texto ressalta a fala de Zaira no tocante ao fato de o Brasil contar apenas com cinco escolas de enfermagem, as quais formavam em média 35 enfermeiras anualmente, e as visitas de Zaira aos hospitais e escolas, no intuito de estudar novos métodos de ensino de enfermagem^(25)^.

Cabe ressaltar que Zaira Cintra Vidal, ao ressaltar a existência de apenas cinco escolas, considerou somente aquelas equiparadas ao modelo de ensino padrão no país. Não obstante, Zaira se fazia presente em outras instituições de ensino de enfermagem, bem como participava de bancas para avaliação de enfermeiros práticos e validação dos diplomas^(21,24)^.

O detalhamento das visitas de Zaira às unidades hospitalares e às escolas de enfermagem no exterior integra o “Relatório dos Estudos feitos na América do Norte”, concluído em 1944. Nesse, constam todos os pontos relevantes de cada instituição visitada. Certamente, o conhecimento adquirido por Zaira constituiu conteúdo importante para a formulação da Lei nº 775, de 6 de agosto de 1949, que tratava do ensino de enfermagem no país. Esse assunto foi objeto de discussão nas reuniões de diretoras de escolas de enfermagem, onde Zaira participava na qualidade de presidente da ABED, sendo também diretora de escola^(24)^.

Na gestão de Zaira como presidente da ABED, foi realizado, em São Paulo, no período de 17 a 22 de março de 1947, o primeiro Congresso Nacional de Enfermagem (hoje denominado Congresso Brasileiro de Enfermagem). A organização da programação do evento coube à Zaira Cintra Vidal e à enfermeira Ella Hasenjaegem, na qualidade de presidente da comissão organizadora, além de Edith de Magalhães Fraenkel e da irmã de caridade Marie Domineuc^(26)^.

Zaira Cintra Vidal, durante o período de 1943 a 1947, ocupou-se também da criação e implantação do projeto de criação da EERHL, atual Faculdade de Enfermagem da UERJ. Cabe informar que a escola foi criada em 1944, mas a inauguração ocorreu em 1948. A equiparação se deu em 1949. Zaira Cintra Vidal ocupou o cargo de diretora de 1944 a 1954^(22)^.

 Para além do curso de graduação em enfermagem, Zaira foi pioneira em coordenar os dois primeiros cursos de pós-graduação para professor em enfermagem, com duração de dois anos (1955 a 1956), e de chefia hospitalar, com duração de um ano (1956)^(22)^. A Figura 3 mostra a fotografia de formatura da primeira turma do curso de professoras em enfermagem da EERHL em 1956.

**Figura 3:** Formatura do curso de pós-graduação em professora de enfermagem da Escola de Enfermeiras Rachel Haddock Lobo

As 19 formandas estão uniformizadas e utilizam capelo. Na composição fotográfica, estão presentes 21 mulheres, todas enfermeiras. No primeiro plano da composição, sentadas, constam dez enfermeiras, sendo a quinta pessoa da esquerda para a direita Sylvia Arcoverde Albuquerque Maranhã, em traje civil, então diretora da EERHL, que tem à sua esquerda Zaira Cintra Vidal, também em traje civil^(27)^.

Zaira Cintra Vidal também atuou junto aos internos do Presidio Frei Caneca, no sentido de promover educação, pois ela comungava com ideias contra o analfabetismo, adotando máxima iluminista surgida no século XIX, qual seja “abrir escolas é fechar prisões”. Portanto, Zaira foi uma enfermeira presente em distintos cenários da sociedade brasileira.

## DISCUSSÃO

Zaira Cintra Vidal, nascida no começo do século XX, era uma mulher não branca cuja classe social era apontada como média^(28)^. Em sua trajetória acadêmica, logrou êxito em ingressar em uma escola de enfermeiras em que foi bem-sucedida, tendo feito estudos de pós-graduação no exterior e se tornado docente dessa mesma escola, em cargos de destaque. Vale refletir que havia decorrido menos de meio século da abolição da escravidão e, certamente, as consequências assolavam sobremaneira os que foram escravizados e suas descendências. Sendo assim, a inserção de Zaira na escola e sua trajetória bem-sucedida evidenciam sua significativa capacidade de estudo e trabalho, pois, no começo do século, a escola nos moldes de Nightingale se certifica de uma boa seleção, admitindo as “moças de boa família”, ou seja, as de pele branca, para caracterizar a imagem da nova profissão na sociedade brasileira^(28,29)^.

O fato é que a mobilidade social da população preta e miscigenada na década de 1920 era difícil, pois, em face das condições das famílias ascendentes, pouco acesso tinham à escolarização, principalmente as mulheres pretas^(28)^. Tal situação reverbera nos anos atuais e, certamente, era muito pior naquela época. Nesse contexto, trazendo para a atualidade, Zaira seria lida socialmente como parda. Assim, o conhecimento e a competência a permitiram ter sua mobilidade social^(29)^.

Para além da atuação como professora, Zaira teve participação importante na difusão do conhecimento científico, por meio da participação expressiva na criação da revista Annaes de Enfermagem, no período de 1930 a 1932. Com o falecimento de Rachel Haddock Lobo, em 26 de setembro de 1933, Zaira Cintra Vidal, então vice-presidente da ABED, assume o cargo de redatora-chefe, em 23 de outubro de 1933, por unanimidade de votos, em uma reunião geral da associação. Nesse mesmo dia, foi estabelecido o dia 25 de novembro para saída do segundo número. As primeiras páginas foram dedicadas à memória de Rachel Haddock Lobo. E no final do ano de 1938, na ocasião de uma nova eleição para a associação, Zaira declarou não pode mais continuar em face de suas inúmeras atividades.

Zaira, no período em que foi redatora-chefe da revista, deu início à sua jornada como escritora de livros para enfermagem. Os livros versavam sobre técnicas de enfermagem da época. Traziam todo um detalhamento dos procedimentos de enfermagem, consoantes com uma linguagem técnica e científica. Os procedimentos eram postos em ordem de complexidade, iniciando do mais simples para o mais complexo. Era um livro, portanto, didático, de fácil leitura e assimilação. No final de cada técnica, possuía um espaço denominado “apontamentos”. Isso demonstrava que Zaira era uma pessoa aberta a receber contribuições, de forma a avançar nas reflexões. Isso evidenciava o seu entendimento que o conhecimento é algo dinâmico e que deve ser compartilhado de forma horizontal^(30)^.

Assim, Zaira democratizou o conhecimento com um material didático em português, dando acesso às pessoas que não compreendiam o inglês. Ademais, era uma obra baseada no pensamento da enfermagem moderna. Sobre publicação de livro, anteriormente aos de Zaira Cintra Vidal, havia o livro escrito pelo médico e oficial do exército Getúlio F. dos Santos denominado “O livro do enfermeiro e da enfermeira”, o qual era destinado àqueles que se dedicavam ao cuidado dos enfermos, no exercício das atividades laborais, na qualidade de enfermeiro ou enfermeira. O livro foi editado em 1916^(31)^. Portanto, Zaira foi a primeira enfermeira brasileira a sistematizar as técnicas de enfermagem em um livro. Além disso, transformou, com o seu conhecimento científico, as técnicas para a realidade brasileira^(13)^.

O livro de Zaira foi dedicado e prefaciado por Rachel Haddock Lobo. Essa estratégia, certamente, capitalizou à escritora e sua obra visibilidade e credibilidade, pois Rachel Haddock Lobo era figura prestigiada no campo da educação e da saúde brasileira, com destacada atuação na EEAN e na ABED. Vale dizer que, por ocasião de sua morte, o cientista Carlos Chagas proferiu discurso prestigioso à beira de seu túmulo, conclamando às enfermeiras a seguirem o seu exemplo^(8)^.

É importante destacar que a pós-graduação de Zaira Cintra Vidal lhe deu competências para assumir cargos de liderança nas áreas da educação e da saúde. Não obstante, apesar de toda a trajetória exitosa na EEAN, não chegou ao cargo de diretora da escola, apesar de atuações interinas e no comando da escola, em cargo correspondente, na atualidade, ao de vice-diretora. Contudo, na ABED, foi eleita por seus pares, para o cargo de presidente, em dois mandatos consecutivos.

Além da edição de livro, em sua atuação na ABED, criou sessões da associação em estados brasileiros onde havia escolas de enfermagem. Tal direcionamento tem caráter inovador para a profissão, pois fortaleceu a associação ao mesmo tempo em que assegurou a formação de grupos capazes de liderar a profissão, de modo a avançar na disseminação do conhecimento e realizações para a enfermagem brasileira. Além disso, foi em sua gestão que houve a realização do primeiro Congresso Nacional de Enfermagem, conhecido hoje como Congresso Brasileiro de Enfermagem. A realização de congresso em âmbito nacional simboliza a constituição de um primeiro espaço intelectual para a profissão, propiciando intercâmbio, difusão do conhecimento e visibilidade. Sendo assim, a visão de vanguarda de Zaira, em prol do desenvolvimento da profissão, em especial no campo da educação, traz à tona, nos anos 1040 e 1950, inovação para as futuras enfermeiras, uma vez que o aprendizado das formas de cuidar envolve estudo e pesquisa, ou seja, “inovações/mudanças a serem instituídas”^(32)^.

Vele pontuar que o congresso e a revista representaram os primeiros espaços intelectuais da profissão. Além de contribuírem para o intercâmbio e avanço do conhecimento, oportunizavam a visibilidade da profissão no cenário da sociedade brasileira, especialmente no tocante à sua vinculação com a produção de conhecimento científico, tão necessária ao avanço da enfermagem e à promoção de um cuidado seguro. Assim, Zaira foi protagonista de uma visão inovadora, no sentido de reconhecer a importância da produção e difusão do conhecimento científico da enfermeira e, consequentemente, da saúde^(33)^. Esses feitos reverberam no tempo presente, quando nos deparamos com o congresso, já em uma 74ª versão, e como a revista, com importante fator de impacto na comunidade científica.

O reconhecimento do trabalho de Zaira na área assistencial, especialmente na organização dos serviços de saúde dos hospitais e na capacitação do pessoal de enfermagem da prefeitura do Rio de Janeiro, projetou-a para ser escolhida para planejar a EERHL, que a levou ao cargo de diretora. Por toda competência profissional de Zaira, a escola foi equiparada à escola padrão em menos de um ano do seu funcionamento^(34)^. Na época, Zaira abriu as portas da escola para o ingresso de meninas pretas, permitindo a mobilidade social das meninas que enfrentavam uma barreira para ingressar no ensino de enfermagem^(29)^.

Além da capacitação do pessoal da Prefeitura do Distrito Federal e do curso de enfermagem da EERHL, em 1955, foram iniciados os cursos de pós-graduação da EERHL nos moldes americanos, em uma época que ainda não existia regulamentação no país, o que viria a acontecer somente na década de 1960^(35)^. Portanto, eis aqui sua vanguarda e a capacidade de inovar a formação do enfermeiro por meio de estudos de pós-graduação.

Portanto, percebendo a inovação em enfermagem como sinônimo de mudança, é pertinente afirmar que Zaira Cintra Vidal inovou a profissão, pois pensou e agiu além do tradicional, do comum, do que estava determinando, para o novo^(36)^. Pode-se dizer que, na primeira metade do século XX, Zaira ousou como visionária e protagonista de mudanças, principalmente na educação em enfermagem vinculada à nascente produção científica.

### Limitações do estudo

Os pesquisadores não conseguiram contatar fontes orais que conviveram com Zaira enquanto familiar, amigos ou alunas, sendo uma fragilidade na construção bibliográfica, pois poderia enriquecer com detalhes subjetivos do agir e pensar de Zaira, tornando o estudo menos linear. Para isso, triangularam-se fontes de diferentes meios de produção e natureza para gerar os resultados a serem analisados e interpretados.

### Contribuições para as áreas da enfermagem, saúde ou políticas públicas

O estudo contribui para preservação da memória da história de vida de Zaira Cintra Vidal, um ícone para a educação em enfermagem e para a saúde, preenchendo lacunas do conhecimento a respeito da história da enfermagem, principalmente no que tange à ABEn, para consolidação da REBEn e para regulamentação do ensino de enfermagem no país.

## CONSIDERAÇÕES FINAIS

Zaira Cintra Vidal teve uma atuação inovadora na enfermagem brasileira. As contribuições dessa inovação demarcam muitos ganhos no tempo presente, os quais podem ser exemplificados pelos congressos ininterruptos, a solidez da REBEn e as seções da ABEn nos diferentes estados do Brasil.

Portanto, Zaira foi baluarte para a profissão no ensino, na pesquisa, na assistência e na vida associativa. Ainda como reflexões de sua atuação, Zaira deixa importante legado para a população feminina e “não branca”, como denominado à época, por meio da consagração de uma trajetória exitosa por meio dos cargos ocupados tanto na ABEn como nas escolas de enfermagem renomadas do então distrito federal. Tais atuações refletem sua capacidade de estudo, trabalho e força para lutar pela participação da mulher em espaços públicos importantes.

## Data Availability

Os dados de pesquisa só estão disponíveis mediante solicitação.
